# Isoliquiritigenin Attenuates Anxiety-Like Behavior and Locomotor Sensitization in Rats after Repeated Exposure to Nicotine

**DOI:** 10.1155/2020/9692321

**Published:** 2020-03-15

**Authors:** Yuhua Wang, Sang Chan Kim, Tong Wu, Yu Jiao, Haifeng Jin, Bong Hyo Lee, Chul Won Lee, Yu Fan, Hee Young Kim, Chae Ha Yang, Zhenglin Zhao, Rongjie Zhao

**Affiliations:** ^1^Department of Psychopharmacology, Qiqihar Medical University, Qiqihar 161006, China; ^2^Medical Research Center, College of Oriental Medicine, Daegu Haany University, Daegu 706-060, Republic of Korea; ^3^Department of Biochemistry, Qiqihar Medical University, Qiqihar 161006, China

## Abstract

As important components of positive and negative reinforcement, locomotor sensitization and withdrawal anxiety following repeated exposure to nicotine (NIC) constitute crucial risk factors for relapse to NIC use after abstinence. *Glycyrrhiza radix* (*G. radix*), an important tonic used in traditional Oriental medicine, has not only anxiolytic effects but also reduces NIC-induced locomotor sensitization. Isoliquiritigenin (ISL), a bioactive ingredient of *G. radix*, also exhibits neuropharmacological effects, including anxiolytic action. Previously, we reported that ISL suppressed cocaine-induced extracellular dopamine release in the nucleus accumbens shell (NaccSh) and attenuated methamphetamine-induced neurotoxicity. The present study was performed to evaluate the effects of ISL on both NIC withdrawal anxiety and locomotor sensitization. Adult male rats received subcutaneous administration of NIC hydrogen tartrate (0.4 mg/kg, twice a day) for 7 days followed by 4 days of withdrawal. During the period of NIC withdrawal, the rats received four intragastric treatments with ISL (3, 10, or 30 mg/kg/day). All three doses of ISL significantly inhibited NIC withdrawal-induced anxiety-like behaviors in the elevated plus maze (EPM) test, but only the 10 mg/kg/day and 30 mg/kg/day ISL doses attenuated locomotor sensitization induced by a challenge dose of NIC. Intracerebroventricular ISL also inhibited both NIC-induced withdrawal anxiety and locomotor sensitization, but intra-NaccSh injection of ISL blocked only NIC locomotor sensitization, which was abolished by post-ISL infusion of *tert*-butyl hydroperoxide (an oxidant) or *N*-methyl-d-aspartate (NMDA) into the NaccSh. Moreover, there was increased protein expression of phosphorylated Erk1/2 in the NIC-sensitized NaccSh, which was suppressed by ISL. Taken together, these results suggest that ISL can inhibit repeated NIC-induced withdrawal anxiety and locomotor sensitization, and the latter is mediated by antagonizing accumbal reactive oxygen species and NMDA receptor signaling.

## 1. Introduction

Tobacco smoking is strongly linked to respiratory disease, cardiovascular disease, diabetes mellitus, and various cancers, and despite a great deal of effort, attempts to quit smoking often end in failure due to nicotine (NIC) dependence [1]. Accordingly, treatment of NIC dependence has become the key factor in quitting smoking. However, with the exception of NIC receptor-based replacement therapies and bupropion, which have shown limited effectiveness [2], no effective pharmacological interventions have yet been reported to aid in overcoming NIC dependence, highlighting the need to develop new pharmaceutical candidates for this purpose.

NIC is strongly addictive, which is sustained by both positive (rewarding effects) and negative reinforcement (withdrawal symptoms). Like other psychostimulants, NIC acts on the mesolimbic dopamine system leading to an increase in dopamine release in the nucleus accumbens shell (NaccSh) to produce rewarding effects, which are behaviorally manifested by increased locomotor activity in rodents, and the higher locomotor activity usually reflects the potency of the reward effect [3, 4]. Repeated NIC exposure escalates locomotor activity in rats; particularly, a challenge dose of NIC evokes much more enhanced locomotor response after withdrawal, a phenomenon referred to as locomotor sensitization [4, 5]. This behavioral sensitization appears to mimic the heightened smoking euphoria after some period of abstinence in smokers and serves as a behavioral marker for the positive reinforcement in NIC dependence and is useful in screening possible pharmacological agents for NIC dependence.

Similar to other major drugs of abuse, abstinence from repeated NIC treatment produces somatic and affective withdrawal symptoms, such as gastrointestinal discomfort, bradycardia, irritability, anxiety, and depression [6], which construct the source of negative reinforcement, driving abstinent smokers to relapse to smoking to relieve the withdrawal discomfort [7]. Among the NIC withdrawal symptoms, anxiety has been identified as the greatest concern because it is the most common withdrawal symptom in abstinent smokers [8] and has been well established in a variety of animal models [9]. Therefore, pharmacologically preventing or relieving anxiety during NIC withdrawal is a promising way to help smokers to quit.

Repeated NIC exposure causes adaptive alterations in the reward circuits to change the patterns of physiological responses to internal and external stimuli, such as augmented response to a challenge dose of drugs of abuse, decreased reward neurotransmission [10], and elevated activities of the stress systems, which underlie both the sensitized behavioral response and withdrawal syndrome [3, 11]. These allostatic changes involve diverse brain reward circuits, fundamentally taking place at the level of neurotransmitters, which ultimately become the targets for pharmacotherapies [3, 12]. Elevated glutamatergic transmission in the NaccSh during NIC withdrawal contributes to both positive [13] and negative reinforcement [9, 14, 15]. Recent evidence has indicated that increased levels of reactive oxygen species (ROS) by repeated exposure to drugs of abuse in several brain regions, including the NaccSh, are also involved in NIC behavioral sensitization [5] and withdrawal anxiety [16]. These findings indicate that bioactive agents that can concurrently antagonize central glutamatergic transmission and oxidative stress represent optimal candidates for pharmacotherapies for NIC dependence.

*Glycyrrhiza radix (G. radix)* is historically used in the treatment of various injuries and detoxification in traditional Oriental medicine due to its well-known anti-inflammatory and antioxidative properties [17], and animal studies over the past two decades have shown that *G. radix* also has neuropharmacological properties, such as neuroprotection and sedation [18]. Especially, *G. radix* was shown to have therapeutic effects on psychostimulant dependence. *G. radix* suppressed acute cocaine-induced dopamine release in the NaccSh [19], inhibited methamphetamine-induced locomotor sensitization in rats [4], and blocked NIC-induced locomotor sensitization by counteracting accumbal oxidative stress [5]. *G. radix* contains various flavonoids and pentacyclic triterpene saponins as the major bioactive constituents, including liquiritigenin, isoliquiritigenin (ISL), liquiritin apioside, and glycyrrhizin [17]. Among these constituents, accumulating evidence suggests that ISL is responsible for the effects of *G. radix* on drug dependence, as ISL shares almost the same pharmacological spectrum with *G. radix* [20]. For example, both were shown to inhibit acute cocaine-induced accumbal dopamine release in the same study [19] and exhibited anxiolytic effects in rats [21, 22]. Similar to *G. radix*, ISL exerts neuroprotective effects via its antioxidant actions [5, 23]. Moreover, ISL is an important phytochemical that antagonizes glutamatergic *N*-methyl-d-aspartate (NMDA) receptors, which improved glutamate-induced cell death of primary cultured rat cortical neurons [24, 25]. Our previous studies also showed that ISL protected against methamphetamine-induced neurotoxicity in the striatum of mice via suppression of glial cell activation [26]. Taken together, these observations suggest that ISL may inhibit both the positive and negative reinforcement induced by repeated NIC use, suggesting its potential for the treatment of NIC dependence.

To examine this possibility, we evaluated the effects of ISL on both repeated NIC-induced withdrawal anxiety and locomotor sensitization in rats and investigated the underlying mechanisms with a focus on the NaccSh.

## 2. Materials and Methods

### 2.1. Reagents

ISL was provided by Shanghai Yuanye Biotechnology Co., Ltd. (Shanghai, China). (−)-Nicotine hydrogen tartrate, *tert*-butyl hydroperoxide (*t*-BOOH), and NMDA were purchased from Sigma-Aldrich (St. Louis, MO, USA). Primary antibodies against total extracellular regulated protein kinases 1/2 (Erk1/2), phospho (P)-Erk1/2, and *β*-actin were obtained from Abcam (Cambridge, UK), and horseradish peroxidase-conjugated secondary antibody was purchased from Cell Signaling Technology (Beverly, MA, USA).

### 2.2. Animals and Experimental Protocols

Nine-week-old male Sprague Dawley rats (280–300 g) were provided by the Laboratory Animal Center at Qiqihar Medical University (Qiqihar, China). The rats were caged three to a group with free access to food and water in an environment with filtered pathogen-free air at a temperature of 21–23°C and relative humidity of 50%, with a 12 : 12 hour light/dark cycle. All experimental procedures adhered to the National Institutes of Health Guide for the Care and Use of Laboratory Animals and were approved by the Animal Care and Use Committee of Qiqihar Medical University (approval number: QMU-AECC-2016-16).

**Figure 1:**
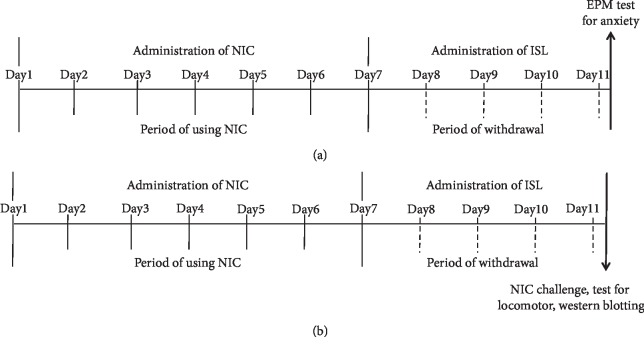
Time schedules for NIC withdrawal (a) and locomotor sensitization (b).

To induce NIC withdrawal, a cohort of rats received subcutaneous injection of 0.4 mg/kg nicotine hydrogen tartrate dissolved in saline (pH 7.2; equal to 0.14 mg/kg NIC free base) twice a day for 7 days in their home cages followed by 4 days of withdrawal. During the NIC withdrawal period, the rats were given ISL intragastrically (3, 10, or 30 mg/kg/day, dissolved in 5% Tween-80) or vehicle once a day for 4 days. At 30 min after the final dose of intragastric ISL, the rats were checked in the elevated plus maze (EPM) paradigm to evaluate anxiety-like behaviors. The experimental groups for evaluating anxiety-like behaviors were as follows: (1) Saline/Vehicle (5% Tween-80) (*n* = 8); (2) NIC/Vehicle (*n* = 8); (3) NIC/ISL03 (3 mg/kg ISL) (*n* = 8); (4) NIC/ISL10 (*n* = 8); and (5) NIC/ISL30 (*n* = 8). To generate NIC locomotor sensitization, another cohort of rats given the same NIC withdrawal schedule and ISL treatments was put into the locomotor testing boxes immediately following the final dose of ISL. A 30 min adaptation period was followed by challenge with 0.4 mg/kg NIC hydrogen tartrate. The animals were left in the boxes for an additional 60 min, and locomotor activities were assessed (Figure 1). The experimental groups for measuring locomotor activities were as follows: (1) Saline/Vehicle/Saline (*n* = 8); (2) Saline/Vehicle/NIC (*n* = 8); (3) NIC/Vehicle/NIC (*n* = 8); (4) NIC/ISL03/NIC (*n* = 8); (5) NIC/ISL10/NIC (*n* = 8); (6) NIC/ISL30/NIC (*n* = 8); and (7) Saline/ISL30/Saline (*n* = 8).

### 2.3. EPM Test

At 30 min after the final dose of ISL, the rats were checked in the EPM to assess anxiety-like behaviors, as described previously [12]. Briefly, the EPM was comprised of two open arms (50 cm long × 10 cm wide) and two closed arms (enclosed by dark acrylic walls 40 cm high), which were elevated above the floor to a height of 50 cm, and monitored with a video-tracking system (Shanghai Xinruan Technology Co., Shanghai, China). At the beginning of the test, each rat was placed on the cross area of the arms, and the numbers of entries into the arms and the time spent by the rats in each arm were recorded over a period of 5 min. The percentages of the number of entries into the open arms and time spent in the open arms were calculated as follows:

(1) $$\begin{array}{c} \%{\text{Entry}}_{\text{open arms}}\;=\;\frac{{\text{Entry}}_{\text{open arms}}}{{\text{Entry}}_{\text{open arms}}\;+{\text{ Entry}}_{\text{closed arms}}}\times 100\%,\\ \%{\text{Time}}_{\text{open arms}}\;=\;\frac{{\text{Time}}_{\text{open arms}}}{{\text{Time}}_{\text{open arms}}+{\text{Time}}_{\text{closed arms}}}\times 100\%. \end{array}$$

### 2.4. Locomotor Activity Test

Locomotor activity was determined in a rectangular box (60 × 60 × 50 cm^3^) with floor and walls made of clear acrylic and painted black. The chamber was equipped with a video camera above the center of the floor, and all locomotor activity of rats was recorded and analyzed by a video-tracking system (Shanghai Xinruan Technology Co.).

### 2.5. Intracerebroventricular (ICV) and Local Microinfusions of ISL

To determine the central and local effects of ISL on NIC dependence, a unilateral injection cannula targeting the right lateral cerebral ventricle and bilateral guide cannulae targeting the NaccSh were implanted under pentobarbital (50 mg/kg, intraperitoneal) anesthesia using a stereotactic instrument (Kopf Instruments, Tujunga, CA, USA). Stereotactic coordinates were as follows: right lateral cerebral ventricle, anterior-posterior (AP) −0.9 mm relative to bregma, medial-lateral (ML) −1.2 mm relative to bregma, dorsal-ventral (DV) −3.5 mm relative to bregma, and NaccSh, AP 1.7 mm, ML ± 0.8 mm, and DV −7.4 mm relative to bregma, according to the Atlas of Paxinos and Watson [27]. Following surgery, the rats were kept in individual cages and given antibiotics and analgesic for 3 days to prevent possible infection and pain. The rats were allowed 7 days to recover from the surgery and underwent the same NIC withdrawal schedule.

During the withdrawal period, ICV infusions of 15 *μ*g/10 *μ*L ISL were conducted once a day for 4 days via a 28-gauge injector using a motorized microsyringe over a period of 90 s. ISL was dissolved in dimethyl sulfoxide and further diluted in modified Ringer's solution (MRS) containing 150 mM NaCl, 3.0 mM KCl, 1.4 mM CaCl_2_, and 0.8 mM MgCl_2_ in 10 mM phosphate buffer (pH 7.2). Five minutes after the fourth ICV ISL administration, the rats were tested in the EPM or challenged by NIC and checked in the locomotor testing boxes. Immediately after the behavioral test, the rats were euthanized and the entire brain was collected. Tissue samples from the NaccSh were punched out for western blotting analyses.

To evaluate whether the effect of ISL on NIC dependence requires the NaccSh, acute bilateral intra-NaccSh microinfusions of ISL (0.5 *μ*g/0.2 *μ*L for each side) were performed 4 days after the final NIC treatment, and 5 min later, the rats were tested in the EPM or challenged with NIC and then checked in the locomotor testing boxes. Similarly, to further identify the possible involvement of accumbal ROS or NMDA receptor pathways in the effects of local ISL on NIC locomotor sensitization, bilateral intra-NaccSh administration of *t*-BOOH (3.0 *μ*g/0.2 *μ*L for each side, dissolved in MRS) or NMDA (0.2 *μ*g/0.2 *μ*L for each side, dissolved in MRS) was performed 5 min after intra-NaccSh ISL, and 5 min later, the rats were challenged by NIC and checked for locomotor activity.

### 2.6. Western Blotting Analysis

NaccSh tissues were homogenized in lysis buffer (20 mM Tris, 5 mM EDTA, and 1% Nonidet P-40 (vol/vol)) containing a protease and phosphatase inhibitor cocktail (Thermo Fisher Scientific Inc., Rockford, IL, USA) and centrifuged at 16,000 × *g* for 20 min at 4°C. The total protein in the supernatants was quantified by bicinchoninic acid assay, separated by electrophoresis, and transferred onto polyvinylidene difluoride membranes (Millipore, Bedford, MA, USA). The membranes were incubated with primary and secondary antibodies, and the corresponding bands of the proteins of interest were visualized using an enhanced chemiluminescence western blot detection kit (Amersham Biosciences, Piscataway, NJ, USA).

### 2.7. Statistical Analysis

All data were analyzed by one-way analysis of variance (ANOVA) followed by Newman–Keuls multiple comparison tests (GraphPad Prism 5.0; GraphPad Software, San Diego, CA, USA) to assess the significance of differences between the experimental groups. All data were expressed as means ± standard errors of the mean (SEM) and analyzed for the normality, and the homogeneity of variances was also checked by Bartlett's test justifying the one-way ANOVA. In all analyses, *p* < 0.05 was taken to indicate statistical significance.

## 3. Results

### 3.1. Effects of Oral ISL on NIC Withdrawal-Induced Anxiety-Like Behavior

In previous studies performed in our laboratory and by other authors, an ISL dose of 20 mg/kg/day was most frequently employed to evaluate its pharmacological effects in rats [19, 23]. A recent report indicated that 30 mg/kg/day ISL for 28 days effectively attenuated monocrotaline-induced pulmonary hypertension in rats without any evident behavioral changes [28]. Moreover, in a preliminary experiment, a single dose of 30 mg/kg ISL ameliorated basal anxiety-like behavior in naive rats (data not shown). Therefore, in this study, ISL doses of 3, 10, and 30 mg/kg/day were selected.

**Figure 2:**
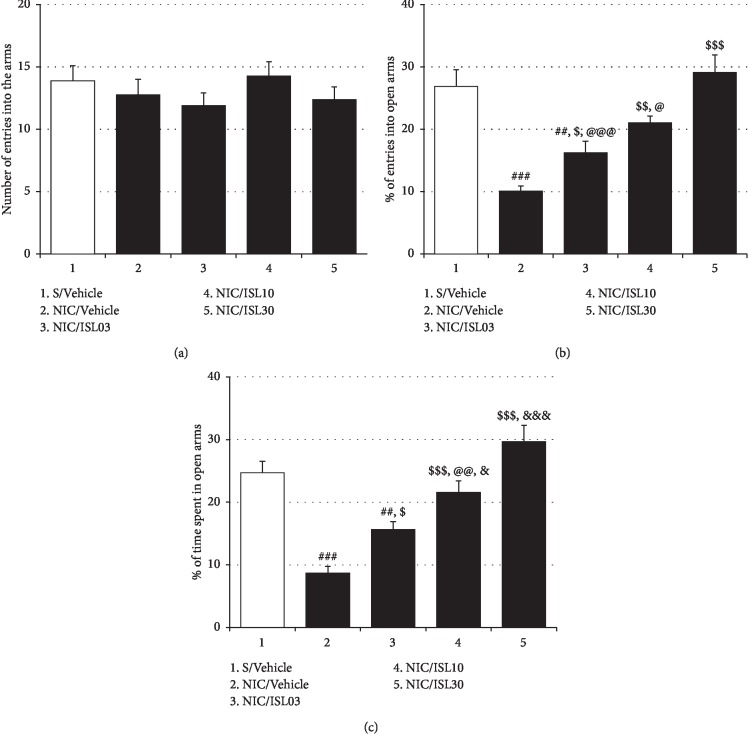
Effects of oral ISL on NIC withdrawal-induced anxiety-like behavior. Withdrawal from repeated NIC treatments resulted in anxiety-like behavior in rats, but these behaviors were mitigated by oral ISL treatment. (a) The total number of entries into open and closed arms of the EPM by rats. (b) The percentage of numbers of entries into open arms of the EPM by rats. (c) The percentage of time spent in open arms by rats. All data are expressed as a mean ± SEM (*n* = 8). S: saline; ISL: isoliquiritigenin; NIC: nicotine; ISL03: 3 mg/kg/d ISL; ISL10: 10 mg/kg/d ISL; ISL30: 30 mg/kg/d ISL. ^##^*p* < 0.01, ^###^*p* < 0.001 vs. S/Vehicle group; ^$^*p* < 0.05, ^$$^*p* < 0.01, ^$$$^*p* < 0.001 vs. NIC/Vehicle group; ^@^*p* < 0.05, ^@@@^*p* < 0.001 vs. NIC/ISL30 group; ^&^*p* < 0.05, ^&&&^*p* < 0.001 vs. NIC/ISL03 group (one-way ANOVA followed by Newman–Keuls post hoc test).

In the present study, NIC withdrawal rats exhibited anxiety-like behavior in the EMP tests when checked 4 days after the last dose of NIC. As shown in Figure 2, NIC withdrawal rats less frequently entered the open arms and spent less time in the open arms than saline-treated controls (%Entry_open arms_: *F*_(4, 35)_ = 14.49, *p* < 0.001; saline-treated control group (Saline/Vehicle) (28.86% ± 2.71%, *n* = 8) vs. NIC-treated control group (NIC/Vehicle) (10.05% ± 0.87%, *n* = 8), *p* < 0.001; %Time_open arms_: *F*_(4, 35)_ = 19.81, *p* < 0.001; Saline/Vehicle group (24.71% ± 1.84%, *n* = 8) vs. NIC/Vehicle group (8.68% ± 1.09%, *n* = 8), *p* < 0.001). However, ISL at all doses examined (3, 10, and 30 mg/kg/day) improved these anxiety indices (%Entry_open arms_: NIC/Vehicle group vs. NIC/ISL03 group (16.22% ± 1.86%, *n* = 8), *p* < 0.05; NIC/Vehicle group vs. NIC/ISL10 group (21.01% ± 1.13%, *n* = 8), *p* < 0.01; NIC/Vehicle group vs. NIC/ISL30 group (29.11% ± 2.84%, *n* = 8), *p* < 0.001; %Time_open arms_: NIC/Vehicle group vs. NIC/ISL03 group (15.64% ± 1.28%, *n* = 8), *p* < 0.05; NIC/Vehicle group vs. NIC/ISL10 group (21.54% ± 1.89%, *n* = 8), *p* < 0.001; NIC/Vehicle group vs. NIC/ISL30 group (29.64% ± 2.63%, *n* = 8), *p* < 0.001), and the effects were dose-dependent (%Entry_open arms_: NIC/ISL03 group vs. NIC/ISL30, *p* < 0.001; NIC/ISL10 group vs. NIC/ISL30 group, *p* < 0.05; %Time_open arms_: NIC/ISL03 group vs. NIC/ISL10 group, *p* < 0.05; NIC/ISL03 group vs. NIC/ISL30 group, *p* < 0.001; NIC/ISL10 group vs. NIC/ISL30 group, *p* < 0.01) (Figure 2).

### 3.2. Effects of Oral ISL on NIC-Induced Locomotor Sensitization

**Figure 3:**
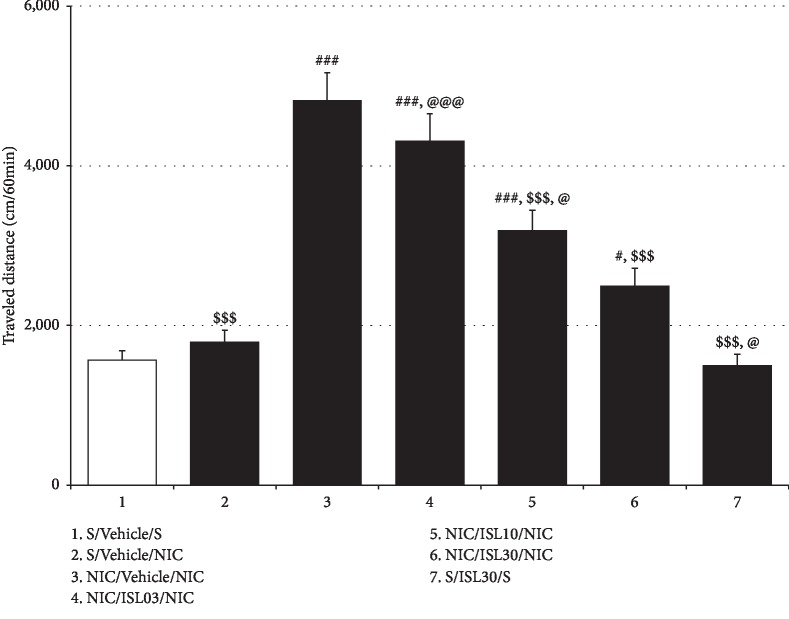
Effects of oral ISL on NIC-induced locomotor sensitization. A NIC challenge 4 days after the termination of repeated NIC administrations induced locomotor sensitization, which was attenuated by oral ISL treatment. All data are expressed as means ± SEM (*n* = 8). S: saline; ISL: isoliquiritigenin; NIC: nicotine; ISL03: 3 mg/kg/d ISL; ISL10: 10 mg/kg/d ISL; ISL30: 30 mg/kg/d ISL. ^#^*p* < 0.05, ^###^*p* < 0.001 vs. S/Vehicle/S group; ^$$$^*p* < 0.001 vs. NIC/Vehicle/NIC group; ^@^*p* < 0.05, ^@@@^*p* < 0.001 vs. NIC/ISL30 group (one-way ANOVA followed by Newman–Keuls post hoc test).

On the 4th day after termination of NIC treatment, NIC challenge generated a significantly greater increase in locomotor activity in NIC-pretreated rats compared to saline-pretreated controls (*F*_(6, 49)_ = 30.41, *p* < 0.001; Saline/Vehicle/Saline group (1565.13 ± 117.62, *n* = 8) vs. NIC/Vehicle/NIC group (4815.63 ± 347.46, *n* = 8), *p* < 0.001) or rats that were given only a challenge dose of NIC (Saline/Vehicle/NIC group (1791.63 ± 147.90, *n* = 8) vs. NIC/Vehicle/NIC group, *p* < 0.001), while the challenge dose alone did not significantly affect locomotor activity (Saline/Vehicle/Saline group vs. Saline/Vehicle/NIC group, *p* > 0.05). These data indicated that repeated NIC treatment induced locomotor sensitization. However, post hoc comparisons showed that ISL at doses of 10 and 30 mg/kg/day during the NIC withdrawal period attenuated the locomotor hypersensitization (NIC/Vehicle/NIC group vs. NIC/ISL10/NIC group (3185.38 ± 257.77, *n* = 8), *p* < 0.001; NIC/Vehicle/NIC group vs. NIC/ISL30/NIC group (2488.75 ± 225.32, *n* = 8), *p* < 0.001) in a dose-dependent manner (NIC/ISL10/NIC group vs. NIC/ISL30/NIC group, *p* < 0.05), while ISL at 3 mg/kg/day did not have a significant effect on locomotor sensitization (NIC/Vehicle/NIC group vs. NIC/ISL03/NIC group (4306.13 ± 347.61, *n* = 8), *p* > 0.05). In addition, post hoc comparisons also showed that 30 mg/kg/day ISL alone did not alter locomotor activity in rats (Saline/Vehicle/Saline group vs. Saline/ISL30/Saline group (1497.75 ± 142.93, *n* = 8), *p* > 0.05) (Figure 3).

### 3.3. Effects of ICV ISL on NIC-Induced Withdrawal Anxiety and Locomotor Sensitization

**Figure 4:**
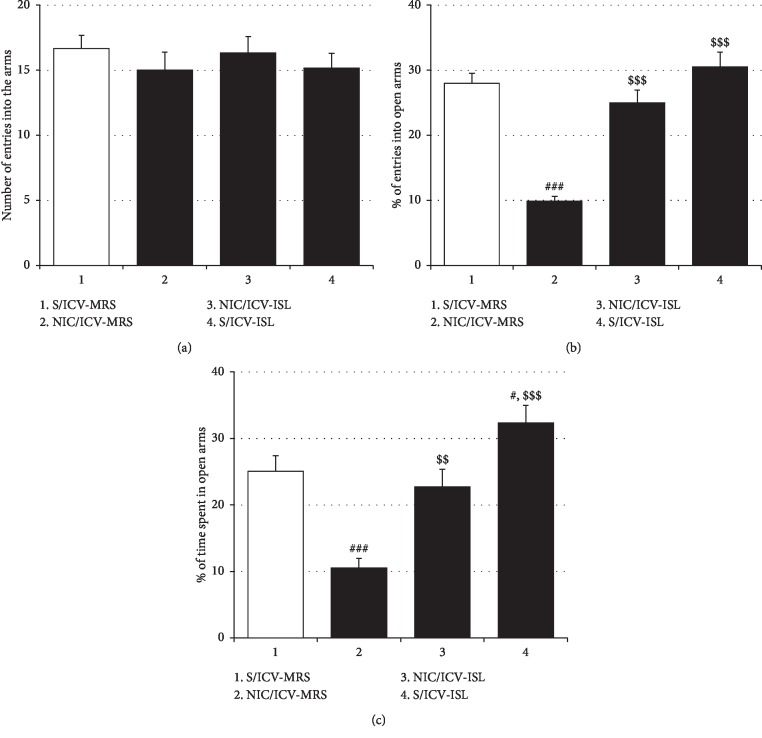
Effects of ICV ISL on NIC-induced withdrawal anxiety. Similar to the case of the oral treatment, ICV ISL treatment during the period of withdrawal ameliorated NIC withdrawal-induced anxiety-like behavior in rats. (a) The total number of entries into open and closed arms of the EPM by rats. (b) The percentage of numbers of entries into open arms of the EPM. (c) The percentage of time spent in open arms. All data are expressed as a mean ± SEM (*n* = 6). S: saline; ISL: isoliquiritigenin; NIC: nicotine; MRS: modified Ringer's solution. ^#^*p* < 0.05, ^###^*p* < 0.001 vs. S/ICV-MRS group; ^$$^*p* < 0.01, ^$$$^*p* < 0.001 vs. NIC/ICV-MRS group (one-way ANOVA followed by Newman–Keuls post hoc test).

**Figure 5:**
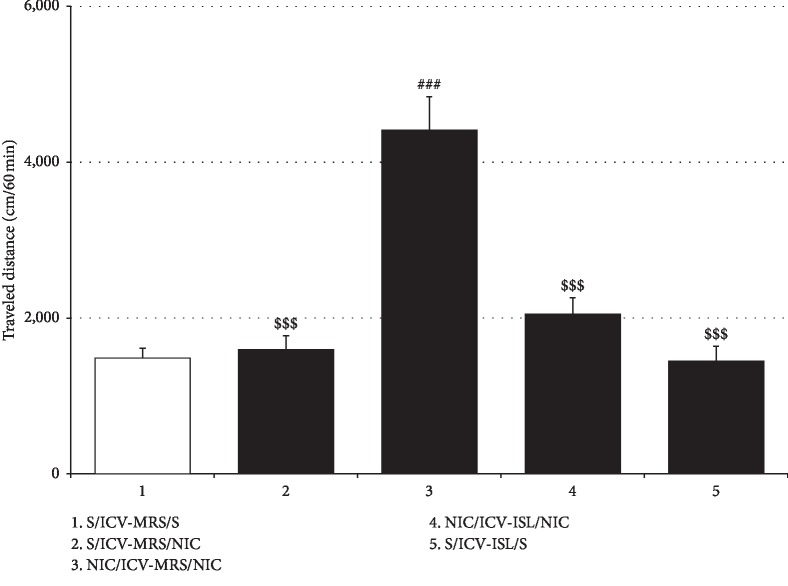
Effects of ICV ISL on NIC-induced locomotor sensitization. Similar to the case of the oral administration, ICV ISL treatment during NIC withdrawal blocked repeated NIC-induced locomotor sensitization. All data are expressed as a mean ± SEM (*n* = 6). S: saline; ISL: isoliquiritigenin; NIC: nicotine; MRS: modified Ringer's solution. ^###^*p* < 0.001 vs. S/ICV-MRS/S group; ^$$$^*p* < 0.001 vs. NIC/ICV-MRS/NIC group (one-way ANOVA followed by Newman–Keuls post hoc test).

Consistent with the effects of oral ISL on NIC-induced anxiety and locomotor sensitization, ICV ISL at a dose of 15 *μ*g/10 *μ*L/day during the 4-day withdrawal period attenuated the respective behavioral changes in rats induced by repeated NIC exposure. As shown in Figure 4, ICV ISL treatment counteracted the reductions in both the number of entries into open arms of the EPM (%Entry_open arms_: *F*_(3, 20)_ = 28.23, *p* < 0.001; Saline/ICV-MRS group (27.97% ± 1.59%, *n* = 6) vs. NIC/ICV-MRS group (9.89% ± 0.73%, *n* = 6), *p* < 0.001; NIC/ICV-MRS group vs. NIC/ICV-ISL group (25.00% ± 1.96%, *n* = 6), *p* < 0.001) and the amount of time spent in the open arms (%Time_open arms_: *F*_(3, 20)_ = 15.25, *p* < 0.001; Saline/ICV-MRS group (20.25% ± 2.34%, *n* = 6) vs. NIC/ICV-MRS group (10.52% ± 1.43%, *n* = 6), *p* < 0.001; NIC/ICV-MRS group vs. NIC/ICV-ISL group (22.72% ± 2.54%, *n* = 6), *p* < 0.001) induced by withdrawal from repeated NIC treatment. ICV ISL treatment alone also significantly increased the time spent in the open arms in saline-pretreated rats, indicating attenuation of innate anxiety in rats (%Time_open arms_: Saline/ICV-MRS group vs. Saline/ICV-ISL group (32.33% ± 2.66%, *n* = 6), *p* < 0.05) (Figure 4). As shown in Figure 5, ICV administration of ISL reduced locomotor distances traveled by NIC-sensitized rats compared to vehicle (MRS) treatment (*F*_(4, 25)_ = 25.31, *p* < 0.001; Saline/ICV-MRS/Saline group (1488.33 ± 122.89, *n* = 6) vs. Saline/ICV-MRS/NIC group (1595.50 ± 179.06, *n* = 6), *p* > 0.05; Saline/ICV-MRS/Saline group vs. NIC/ICV-MRS/NIC group (4409.83 ± 431.15, *n* = 6), *p* < 0.001; Saline/ICV-MRS/NIC group vs. NIC/ICV-MRS/NIC group, *p* < 0.001; NIC/ICV-MRS/NIC group vs. NIC/ICV-ISL/NIC group (2049.17 ± 209.36, *n* = 6), *p* < 0.001). ICV ISL treatment alone did not significantly affect locomotor activity in rats, which was consistent with the data obtained with oral ISL treatment (Saline/ICV-MRS/Saline group vs. Saline/ICV-ISL/Saline group (1444.67 ± 191.43, *n* = 6), *p* > 0.05) (Figure 5).

### 3.4. Effects of Intra-NaccSh Infusion of ISL on NIC-Induced Withdrawal Anxiety and Locomotor Sensitization

**Figure 6:**
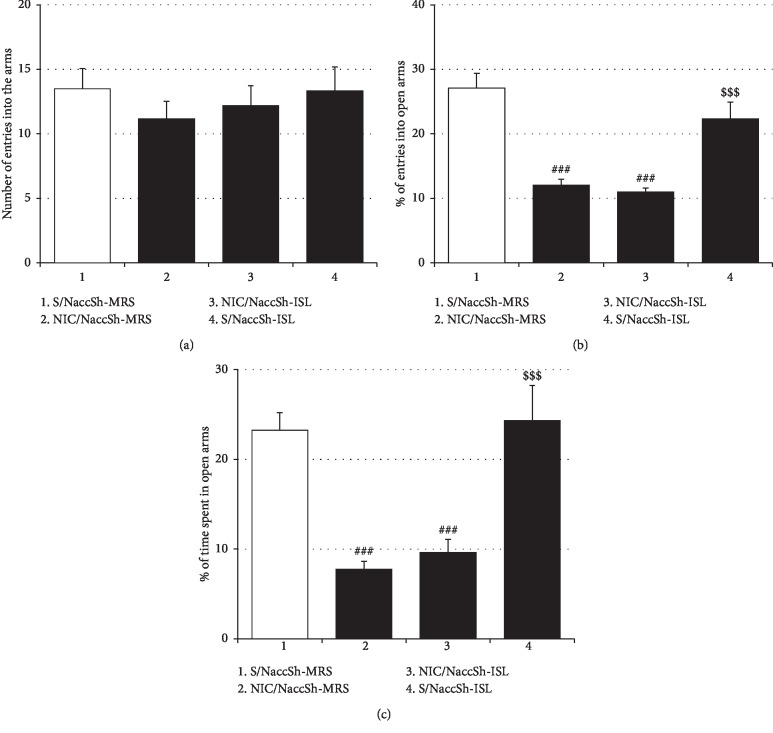
Effects of intra-NaccSh infusion of ISL on NIC withdrawal-induced anxiety. Unlike the oral and the ICV treatment, the intra-NaccSh injection of ISL did not affect NIC withdrawal-induced anxiety-like behavior in rats. (a) The total number of entries into open and closed arms of the EPM by rats. (b) The percentage of numbers of entries into open arms of the EPM. (c) The percentage of time spent in open arms. All data are expressed as a mean ± SEM (*n* = 6). S: saline; ISL: isoliquiritigenin; NIC: nicotine; MRS: modified Ringer's solution. ^###^*p* < 0.001 vs. S/NaccSh-MRS group; ^$$$^*p* < 0.001 vs. NIC/NaccSh-MRS group (one-way ANOVA followed by Newman–Keuls post hoc test).

**Figure 7:**
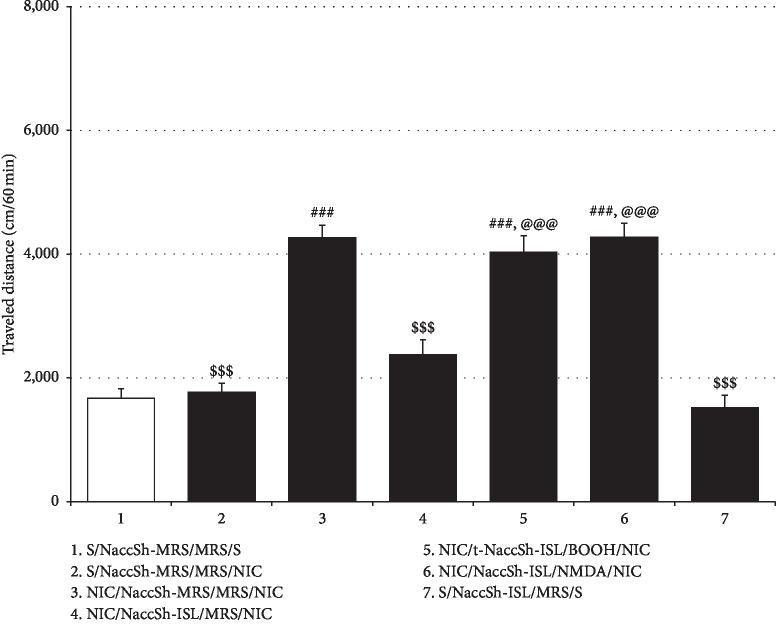
Effects of intra-NaccSh infusion of ISL on repeated NIC-induced locomotor sensitization. Similar to the oral and the ICV treatment, the intra-NaccSh administration of ISL blocked the expression of repeated NIC-induced locomotor sensitization, which was abrogated by a post-ISL infusion of either *t*-BOOH or NMDA into the NaccSh. All data are expressed as a mean ± SEM (*n* = 6). S: saline; ISL: isoliquiritigenin; NIC: nicotine; MRS: modified Ringer's solution. ^###^*p* < 0.001 vs. S/NaccSh-MRS/MRS/S group; ^$$$^*p* < 0.001 vs. NIC/NaccSh-MRS/MRS/NIC group; ^@@@^*p* < 0.001 vs. NIC/NaccSh-ISL/MRS/NIC group (one-way ANOVA followed by Newman–Keuls post hoc test).

Unlike oral and ICV treatments, intra-NaccSh ISL administration failed to alter NIC withdrawal-induced anxiety-like behaviors in rats, as shown in Figure 6 (%Entry_open arms_: *F*_(3, 20)_ = 18.50, *p* < 0.001; Saline/NaccSh-MRS group (27.08% ± 2.33%, *n* = 6) vs. NIC/NaccSh-MRS group (12.06% ± 0.92%, *n* = 6), *p* < 0.001; NIC/NaccSh-MRS group vs. NIC/NaccSh-ISL group (10.99% ± 0.63%, *n* = 6), *p* > 0.05; %Time_open arms_: *F*_(3, 20)_ = 13.78, *p* < 0.001; Saline/NaccSh-MRS group (23.23% ± 2.00%, *n* = 6) vs. NIC/NaccSh-MRS group (7.73% ± 0.92%, *n* = 6), *p* < 0.001; NIC/NaccSh-MRS group vs. NIC/NaccSh-ISL group (9.60% ± 1.48%, *n* = 6), *p* > 0.05); and intra-NaccSh ISL alone exhibited no anxiolytic effect (%Entry_open arms_: Saline/NaccSh-MRS group vs. Saline/NaccSh-ISL group (22.34% ± 2.59%, *n* = 6), *p* > 0.05; %Time_open arms_: Saline/NaccSh-MRS group vs. Saline/NaccSh-ISL group (24.32% ± 3.91%, *n* = 6), *p* > 0.05). However, similar to oral and ICV treatments, intra-NaccSh ISL effectively blocked NIC locomotor sensitization, as shown in Figure 7 (*F*_(6, 35)_ = 37.77, *p* < 0.001; Saline/NaccSh-MRS/MRS/Saline group (1666.67 ± 159.75, *n* = 6) vs. Saline/NaccSh-MRS/MRS/NIC group (1764.67 ± 151.08, *n* = 6), *p* > 0.05; Saline/NaccSh-MRS/MRS/Saline group vs. NIC/NaccSh-MRS/MRS/NIC group (4265.33 ± 205.08, *n* = 6), *p* < 0.001; Saline/NaccSh-MRS/MRS/NIC group vs. NIC/NaccSh-MRS/MRS/NIC group, *p* < 0.001; NIC/NaccSh-MRS/MRS/NIC group vs. NIC/NaccSh-ISL/MRS/NIC group (2370.83 ± 242.65, *n* = 6), *p* < 0.001), which was abolished by post-ISL infusion of *t*-BOOH (NIC/NaccSh-ISL/*t*-BOOH/NIC group (4031.17 ± 268.93, *n* = 6) vs. NIC/NaccSh-ISL/MRS/NIC group, *p* < 0.001; NIC/NaccSh-ISL/*t*-BOOH/NIC group vs. NIC/NaccSh-MRS/MRS/NIC group, *p* > 0.05) or NMDA (NIC/NaccSh-ISL/NMDA/NIC group (4276.33 ± 222.02, *n* = 6) vs. NIC/NaccSh-ISL/MRS/NIC group, *p* < 0.001; NIC/NaccSh-ISL/NMDA/NIC group vs. NIC/NaccSh-MRS/MRS/NIC group, *p* > 0.05) into the NaccSh. Intra-NaccSh ISL treatment alone did not show a significant effect on locomotor activity in rats.

### 3.5. Effects of ICV ISL on Accumbal Erk1/2 Expression and Phosphorylation

**Figure 8:**
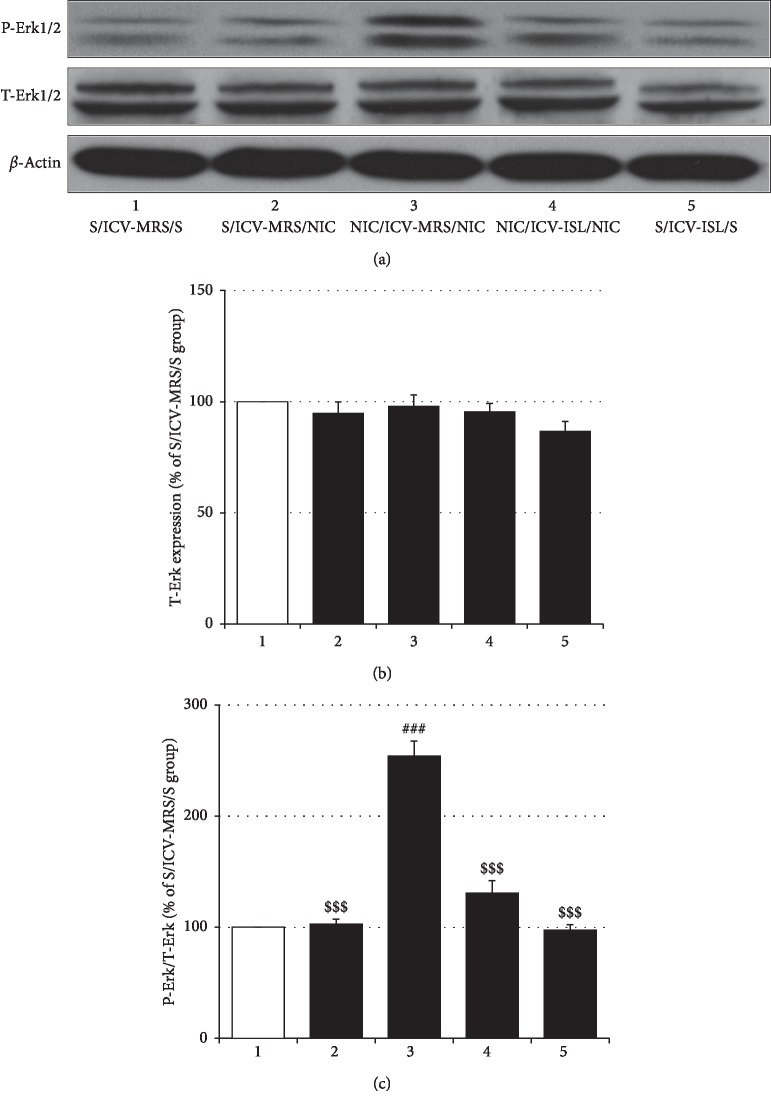
Effects of ICV ISL on accumbal Erk1/2 expression and phosphorylation. A challenge dose of NIC 4 days after termination of repeated NIC treatment did not significantly affect the expression of total Erk1/2 in the NaccSh but elevated its phosphorylation rate, which was normalized by ICV ISL treatment. All data are expressed as a mean ± SEM (*n* = 4). S: saline; ISL: isoliquiritigenin; NIC: nicotine; MRS: modified Ringer's solution. T-Erk1/2: total Erk1/2; P-Erk1/2: phosphorylated Erk1/2. ^###^*p* < 0.001 vs. S/ICV-MRS/S group; ^$$$^*p* < 0.001 vs. NIC/ICV-MRS/NIC group (one-way ANOVA followed by Newman–Keuls post hoc test). (a) T-Erk expression (% of S/ICV-MRS/S group) and (b) P-Erk/T-Erk (% of S/ICV-MRS/S group).

The levels of Erk1/2 protein expression in the NaccSh showed no differences between NIC-sensitized and saline-pretreated rats regardless of challenge with saline or NIC (Erk1/2: *F*_(4, 15)_ = 1.49, *p* > 0.05; Saline/ICV-MRS/Saline group (100.00% ± 0.00%, *n* = 4) vs. NIC/ICV-MRS/NIC (98.98% ± 5.10%, *n* = 4), *p* > 0.05; Saline/ICV-MRS/NIC group (94.78% ± 5.07%, *n* = 4) vs. NIC/ICV-MRS/NIC group, *p* > 0.05), and ICV ISL treatment alone did not influence accumbal Erk1/2 expression (Saline/ICV-MRS/Saline group vs. Saline/ICV-ISL/Saline group (86.59% ± 4.55%, *n* = 4), *p* > 0.05; NIC/ICV-MRS/NIC group vs. NIC/ICV-ISL/NIC group (95.35 ± 3.93, *n* = 4), *p* > 0.05). However, NIC sensitization significantly elevated the phosphorylation of Erk1/2 in the NaccSh (P-Erk1/2: *F*_(4, 15)_ = 64.85, *p* < 0.001; Saline/ICV-MRS/Saline group (100.00% ± 0.00%, *n* = 4) vs. NIC/ICV-MRS/NIC group (254.19% ± 13.16%, *n* = 4), *p* < 0.001; Saline/ICV-MRS/NIC group (102.59% ± 4.56%, *n* = 4) vs. NIC/ICV-MRS/NIC group, *p* < 0.001), while ICV ISL treatment suppressed this elevation (NIC/ICV-MRS/NIC group vs. NIC/ICV-ISL/NIC group (130.69 ± 11.30, *n* = 4), *p* < 0.001). A single administration of NIC or ICV ISL alone did not alter the phosphorylation of Erk1/2 in the NaccSh (Saline/ICV-MRS/Saline group vs. Saline/ICV-MRS/NIC group, *p* > 0.05; Saline/ICV-MRS/Saline group vs. Saline/ICV-ISL/Saline (97.36% ± 4.83%, *n* = 4), *p* > 0.05) (Figure 8).

## 4. Discussion

Previous studies from our research team and other laboratories have suggested that ISL may have therapeutic effects on NIC dependence. Therefore, in the present study, we established models of withdrawal anxiety and locomotor sensitization by exposing rats to repeated NIC treatment and evaluated the effects of ISL in these models.

The results of the present study indicated that treatment with ISL (3, 10, and 30 mg/kg/day) four times during the withdrawal period dose-dependently inhibited repeated NIC-induced withdrawal anxiety, and although 3 mg/kg/day ISL did not significantly alter NIC locomotor sensitization, both 10 and 30 mg/kg/day ISL attenuated the behavioral sensitization in a dose-dependent manner. In agreement with these observations, when given via the ICV route, ISL blocked both withdrawal anxiety and locomotor sensitization. However, in the local injection experiment, the intra-NaccSh ISL administration spared withdrawal anxiety but inhibited behavioral sensitization, which was abolished by post-ISL infusion of *t*-BOOH or NMDA. Moreover, in western blotting assay, ISL normalized the protein expression of P-Erk1/2 in the NIC-sensitized NaccSh. Taken together, these results suggest that ISL can inhibit both withdrawal anxiety and locomotor sensitization induced by repeated NIC treatment, and the effect on behavioral sensitization is mediated via its antioxidant and anti-NMDA receptor signaling actions.

Withdrawal from repeated use of NIC in rodents produces substantial anxiety-like behaviors in various instrumental tests, including the EPM test; these tests have been validated in previous studies in our laboratory and by other groups [12, 29]. As expected, in the present study, the ethological phenotype of anxiety was observed in the EPM test in that the NIC withdrawal rats exhibited decreased numbers of entries into open arms and spent less time in the open arms of the EPM compared to saline-treated controls. However, oral administration of 3, 10, and 30 mg/kg/day ISL reversed the decreased number of entries into open arms and prolonged the time spent in open arms in a dose-dependent manner. These results indicated that oral treatment with ISL during the withdrawal period could improve withdrawal-induced anxiety. It is worth noting that Jamal et al. [22] reported that 25 mg/kg/day ISL for 3 days improved the behavioral scores of anxiety in naive rats in EMP tests. As NIC withdrawal perturbs the central neuroendocrine system to induce a pathophysiological state of anxiety [30], our results along with this report suggest that ISL can attenuate both innate and pathophysiological anxiety.

Repeated exposure of rats to NIC elicited behavioral sensitization [3, 5] as indicated in the present study by the observation that nicotine-pretreated rats traveled greater distances than their saline-pretreated counterparts when challenged with the same dose of NIC. However, this locomotor sensitization was prevented by both 10 and 30 mg/kg/day ISL. These results indicated that ISL treatment during the withdrawal period could impede the development of behavioral sensitization. These observations were consonant with our previous findings, indicating that methanol extracts of *G. radix* containing ISL blocked repeated NIC-induced locomotor sensitization [5], and were also compatible with our other previous study, indicating that ISL curbed acute cocaine-induced phasic release of dopamine in the NaccSh [19]. It should be noted that 3 mg/kg/day ISL failed to affect the development of NIC locomotor sensitization, while the same dose mitigated NIC withdrawal-induced anxiety in the present study. This discrepancy may have been due to differences in the involvement of the central nervous system in the development of NIC locomotor sensitization and NIC withdrawal anxiety and also suggested that ISL may be more efficient in treating NIC withdrawal anxiety than NIC locomotor sensitization.

A flavonoid with a chalcone structure, ISL, readily passes through the blood-brain barrier to exert its central actions [31]. Peripherally injected ISL is metabolized to liquiritigenin (another important bioactive flavonoid in *G. radix*) and other glucuronidated metabolites via cytochrome P450 2C19 and UDP-glucuronosyltransferases in the liver [32, 33]. Liquiritigenin has also been shown to have neuroprotective effects on dysregulation of the central nervous system [34]. Specifically, liquiritigenin was reported to ameliorate acute cocaine-induced hyperlocomotion in rats [35], suggesting that the behavioral effects of oral ISL on NIC dependence may be mediated via its metabolite, liquiritigenin. However, there is evidence that this may not be the case, as Lee et al. [36] detected no liquiritigenin in the rat brain 30 min after intravenous administration of 100 mg/kg ISL. To further determine the central effects of ISL on NIC dependence, ICV administration of ISL was performed in the present study. The results showed that ICV ISL at a dose of 15 *μ*g/day during the NIC withdrawal period not only improved anxiety scores of NIC withdrawn rats but also attenuated NIC locomotor sensitization. These results confirmed that ISL directly produces its anxiolytic effects and antisensitization actions by recruiting some central mechanisms and strongly supported the same effects of oral ISL treatment.

The NaccSh is a key structure in the brain underlying drug-craving behaviors, including NIC locomotor sensitization [3, 37]. In addition, accumulating evidence over the past two decades has shown that the NaccSh also contributes to the negative reinforcement of drug dependence, such as withdrawal anxiety and depression [38, 39]. Therefore, to identify whether the NaccSh is the common substrate for the effects of ISL on NIC withdrawal anxiety and locomotor sensitization, in the present study, intra-NaccSh ISL administration was performed. The results found that bilateral intra-NaccSh injection of ISL effectively abated NIC locomotor sensitization, but the same dose of ISL did not affect NIC withdrawal-induced anxiety-like behavior. As mentioned above, the efficacy of ISL seems to be higher in treating NIC withdrawal anxiety than NIC locomotor sensitization. Therefore, these results suggest that the NaccSh is the brain locus mediating the antisensitization action of ISL, but not the site for the anxiolytic effect of ISL. These results also corroborate our previous report that methanol extracts of *G. radix* attenuated NIC locomotor sensitization via a NaccSh mechanism [19].

The NaccSh receives glutamatergic inputs from various brain regions, such as the prefrontal cortex and the amygdala [40, 41], and altered glutamatergic transmission within the NaccSh plays a key role in mediating addiction-related behaviors, including locomotor sensitization [42]. Repeated exposure to NIC leads to long-term potentiation in the NaccSh that underlies behavioral sensitization, entailing activation of postsynaptic NMDA receptors [43]. The noncompetitive NMDA receptor antagonist, MK-801, inhibits NIC locomotor sensitization [13], and another NMDA receptor antagonist, acamprosate, attenuates morphine-induced behavioral sensitization by reducing extracellular dopamine release in the NaccSh [44]. Meanwhile, ROS within the NaccSh that act as a molecular signal in transmission of the reward effects of drugs of abuse [45] appear to be involved in locomotor sensitization. For example, increased accumbal ROS production is associated with cocaine self-administration and methamphetamine locomotor sensitization [45, 46]. As mentioned above, methanol extracts of *G. radix* have been reported to block NIC locomotor sensitization and the sensitized extracellular dopamine release by normalizing ROS production in the NaccSh. In the present study, the post-ISL infusion experiment also showed that intra-NaccSh infusion of *t*-BOOH or NMDA abolished the antisensitization effect of ISL. These results indicated that the effect of ISL on NIC locomotor sensitization is mediated via antagonism of accumbal oxidative stress and glutamatergic (NMDA receptor) transmission.

Postsynaptic activation of NMDA receptors leads to the entry of Ca^2+^ into neurons to increase the phosphorylation of protein kinases, including Erk1/2 [47]. Cocaine locomotor sensitization induced by repeated cocaine treatment increases NMDA receptor transduction, which is accompanied by elevated P-Erk1/2 in the NaccSh [48]. Elevated ROS production also promotes the phosphorylation of Erk1/2 in neuronal cells [49, 50]. Therefore, increased levels of P-Erk1/2 in NIC-sensitized NaccSh can be considered to represent an integrating biomolecular point of heightened glutamatergic transduction and ROS production. Indeed, in the present study, western blotting showed that NIC sensitization increased P-Erk1/2 in the NaccSh but spared the expression of total Erk1/2. Daily ICV ISL treatment during the NIC withdrawal period blocked the increase in P-Erk1/2 in the NIC-sensitized NaccSh. These results suggested that ISL inhibits repeated NIC-stimulated phosphorylation of accumbal Erk1/2, and support the suggestion that ISL attenuates NIC sensitization by antagonizing accumbal oxidative stress and NMDA receptor signaling.

In summary, the results of the present study indicate that oral ISL treatment dose-dependently ameliorated NIC withdrawal anxiety and attenuated NIC locomotor sensitization in rats. The same effects of ISL were also observed when administered via the ICV route. However, when locally administered, intra-NaccSh ISL did not affect NIC withdrawal anxiety but blocked NIC sensitization, and the latter was abolished by post-ISL infusion of *t*-BOOH or NMDA. Moreover, ICV ISL treatment inhibited the increased phosphorylation of Erk1/2 in the NIC-sensitized NaccSh. These results suggested that ISL can block both the positive and negative reinforcement of repeated NIC use and that it therefore represents a promising pharmacological candidate for the treatment of NIC dependence.

## Data Availability

The data supporting the conclusions of the present study are properly analyzed and included in Results section and are available from the corresponding author upon reasonable request.

## References

[1] West R. (2017). Tobacco smoking: health impact, prevalence, correlates and interventions. *Psychology & Health*.

[2] Dwoskin L. P., Smith A. M., Wooters T. E., Zhang Z., Crooks P. A., Bardo M. T. (2009). Nicotinic receptor-based therapeutics and candidates for smoking cessation. *Biochemical Pharmacology*.

[3] Shim I., Javaid J. I., Wirtshafter D. (2001). Nicotine-induced behavioral sensitization is associated with extracellular dopamine release and expression of c-Fos in the striatum and nucleus accumbens of the rat. *Behavioural Brain Research*.

[4] Zhao Z., Kim Y. W., Yang Y. (2014). Glycyrrhizae radix methanol extract attenuates methamphetamine-induced locomotor sensitization and conditioned place preference. *Evidence-Based Complementary and Alternative Medicine*.

[5] Zhao Z. L., Kim S. C., Liu H. F. (2017). Blockade of nicotine sensitization by methanol extracts of Glycyrrhizae radix mediated via antagonism of accumbal oxidative stress. *BMC Complementary and Alternative Medicine*.

[6] McLaughlin I., Dani J. A., De Biasi M. (2015). Nicotine withdrawal. *The Neuropharmacology of Nicotine Dependence*.

[7] Shiffman S., West R., Gilbert D. (2004). Recommendation for the assessment of tobacco craving and withdrawal in smoking cessation trials. *Nicotine & Tobacco Research*.

[8] Morissette S. B., Tull M. T., Gulliver S. B., Kamholz B. W., Zimering R. T. (2007). Anxiety, anxiety disorders, tobacco use, and nicotine: a critical review of interrelationships. *Psychological Bulletin*.

[9] Molas S., DeGroot S. R., Zhao-Shea R., Tapper A. R. (2017). Anxiety and nicotine dependence: emerging role of the habenulo-interpeduncular axis. *Trends in Pharmacological Sciences*.

[10] Benowitz N. L. (2008). Neurobiology of nicotine addiction: implications for smoking cessation treatment. *The American Journal of Medicine*.

[11] Bruijnzeel A. W. (2017). Neuropeptide systems and new treatments for nicotine addiction. *Psychopharmacology*.

[12] Gu C., Zhao Z., Zhu X. (2018). Aqueous extract of semen ziziphi spinosae exerts anxiolytic effects during nicotine withdrawal via improvement of amygdaloid CRF/CRF1R signaling. *Evidence-based Complementary and Alternative Medicine*.

[13] Shim I., Kim H. T., Kim Y. H. (2002). Role of nitric oxide synthase inhibitors and NMDA receptor antagonist in nicotine-induced behavioral sensitization in the rat. *European Journal of Pharmacology*.

[14] Jain R., Mukherjee K., Balhara Y. P. S. (2008). The role of NMDA receptor antagonists in nicotine tolerance, sensitization, and physical dependence: a preclinical review. *Yonsei Medical Journal*.

[15] Watanabe T., Nakagawa T., Yamamoto R., Maeda A., Minami M., Satoh M. (2002). Involvement of glutamate receptors within the central nucleus of the amygdala in naloxone-precipitated morphine withdrawal-induced conditioned place aversion in rats. *The Japanese Journal of Pharmacology*.

[16] Reckziegel P., Boufleur N., Barcelos R. C. S. (2011). Oxidative stress and anxiety-like symptoms related to withdrawal of passive cigarette smoke in mice: beneficial effects of pecan nut shells extract, a by-product of the nut industry. *Ecotoxicology and Environmental Safety*.

[17] Asl M. N., Hosseinzadeh H. (2008). Review of pharmacological effects of Glycyrrhiza sp. and its bioactive compounds. *Phytotherapy Research*.

[18] Yang R., Wang L.-Q., Yuan B.-C., Liu Y. (2015). The pharmacological activities of licorice. *Planta Medica*.

[19] Jang E. Y., Choe E. S., Hwang M. (2008). Isoliquiritigenin suppresses cocaine-induced extracellular dopamine release in rat brain through GABA_B_ receptor. *European Journal of Pharmacology*.

[20] Peng F., Du Q., Peng C. (2015). A review: the pharmacology of isoliquiritigenin. *Phytotherapy Research*.

[21] Park H.-J., Shim H. S., Kim H. (2010). Effects of Glycyrrhizae radixon repeated restraint stress-induced neurochemical and behavioral responses. *The Korean Journal of Physiology and Pharmacology*.

[22] Jamal H., Ansari W. H., Rizvi S. J. (2008). Evaluation of chalcones—a flavonoid subclass, for, their anxiolytic effects in rats using elevated plus maze and open field behaviour tests. *Fundamental & Clinical Pharmacology*.

[23] Zhu X., Liu J., Huang S. (2019). Neuroprotective effects of isoliquiritigenin against cognitive impairment via suppression of synaptic dysfunction, neuronal injury, and neuroinflammation in rats with kainic acid-induced seizures. *International Immunopharmacology*.

[24] Kawakami Z., Ikarashi Y., Kase Y. (2011). Isoliquiritigenin is a novel NMDA receptor antagonist in kampo medicine yokukansan. *Cellular and Molecular Neurobiology*.

[25] Lee H. K., Yang E.-J., Kim J. Y., Song K.-S., Seong Y. H. (2012). Inhibitory effects of glycyrrhizae radix and its active component, isoliquiritigenin, on A*β*(25–35)-induced neurotoxicity in cultured rat cortical neurons. *Archives of Pharmacal Research*.

[26] Lee M. J., Yang C. H., Jeon J.-P., Hwang M. (2009). Protective effects of isoliquiritigenin against methamphetamine-induced neurotoxicity in mice. *Journal of Pharmacological Sciences*.

[27] Paxinos G., Watson C. (1988). *The Rat Brain in Stereotaxic Coordinates*.

[28] Jin H., Jiang Y., Du F. (2019). Isoliquiritigenin attenuates monocrotaline-induced pulmonary hypertension via inhibition of the inflammatory response and PASMCs proliferation. *Evidence-based Complementary and Alternative Medicine*.

[29] Brynildsen J. K., Lee B. G., Perron I. J., Jin S., Kim S. F., Blendy J. A. (2018). Activation of AMPK by metformin improves withdrawal signs precipitated by nicotine withdrawal. *Proceedings of the National Academy of Sciences*.

[30] Watkins S. S., Koob G. F., Markou A. (2000). Neural mechanisms underlying nicotine addiction: acute positive reinforcement and withdrawal. *Nicotine & Tobacco Research*.

[31] Zou P., Ji H.-M., Zhao J.-W. (2019). Protective effect of isoliquiritigenin against cerebral injury in septic mice via attenuation of NF-*κ*B. *Inflammopharmacology*.

[32] Guo J., Liu D., Nikolic D., Zhu D., Pezzuto J. M., Van Breemen R. B. (2008). In vitro metabolism of isoliquiritigenin by human liver microsomes. *Drug Metabolism and Disposition*.

[33] Guo J., Liu A., Cao H., Luo Y., Pezzuto J. M., Van Breemen R. B. (2008). Biotransformation of the chemopreventive agent 2′,4′,4-trihydroxychalcone (isoliquiritigenin) by UDP-glucuronosyltransferases. *Drug Metabolism and Disposition*.

[34] Yang E.-J., Park G. H., Song K.-S. (2013). Neuroprotective effects of liquiritigenin isolated from licorice roots on glutamate-induced apoptosis in hippocampal neuronal cells. *Neurotoxicology*.

[35] Jang E. Y., Hwang M., Yoon S. S. (2011). Liquiritigenin decreases selective molecular and behavioral effects of cocaine in rodents. *Current Neuropharmacology*.

[36] Lee Y. K., Chin Y. W., Bae J. K., Seo J. S., Choi Y. H. (2013). Pharmacokinetics of isoliquiritigenin and its metabolites in rats: low bioavailability is primarily due to the hepatic and intestinal metabolism. *Planta Medica*.

[37] Di Chiara G. (2000). Role of dopamine in the behavioural actions of nicotine related to addiction. *European Journal of Pharmacology*.

[38] Morud J., Strandberg J., Andrén A., Ericson M., Söderpalm B., Adermark L. (2018). Progressive modulation of accumbal neurotransmission and anxiety-like behavior following protracted nicotine withdrawal. *Neuropharmacology*.

[39] Ren Q., Ma M., Yang C., Zhang J.-C., Yao W., Hashimoto K. (2015). BDNF-TrkB signaling in the nucleus accumbens shell of mice has key role in methamphetamine withdrawal symptoms. *Translational Psychiatry*.

[40] Ma Y.-Y., Lee B. R., Wang X. (2014). Bidirectional modulation of incubation of cocaine craving by silent synapse-based remodeling of prefrontal cortex to accumbens projections. *Neuron*.

[41] McDonald A. J. (1991). Topographical organization of amygdaloid projections to the caudatoputamen, nucleus accumbens, and related striatal-like areas of the rat brain. *Neuroscience*.

[42] Lenoir M., Kiyatkin E. A. (2013). Intravenous nicotine injection induces rapid, experience-dependent sensitization of glutamate release in the ventral tegmental area and nucleus accumbens. *Journal of Neurochemistry*.

[43] Vezina P., McGehee D. S., Green W. N. (2007). Exposure to nicotine and sensitization of nicotine-induced behaviors. *Progress in Neuro-Psychopharmacology and Biological Psychiatry*.

[44] Spanagel R., Sillaber I., Zieglgänsberger W., Corrigall W. A., Stewart J., Shaham Y. (1998). Acamprosate suppresses the expression of morphine-induced sensitization in rats but does not affect heroin self-administration or relapse induced by heroin or stress. *Psychopharmacology*.

[45] Jang E. Y., Ryu Y.-H., Lee B. H. (2015). Involvement of reactive oxygen species in cocaine-taking behaviors in rats. *Addiction Biology*.

[46] Jang E. Y., Yang C. H., Hedges D. M. (2017). The role of reactive oxygen species in methamphetamine self-administration and dopamine release in the nucleus accumbens. *Addiction Biology*.

[47] Xia Z., Dudek H., Miranti C. K., Greenberg M. E. (1996). Calcium influx via the NMDA receptor induces immediate early gene transcription by a MAP kinase/ERK-dependent mechanism. *The Journal of Neuroscience*.

[48] Schumann J., Yaka R. (2009). Prolonged withdrawal from repeated noncontingent cocaine exposure increases NMDA receptor expression and ERK activity in the nucleus accumbens. *Journal of Neuroscience*.

[49] Wu H., Ichikawa S., Tani C. (2009). Docosahexaenoic acid induces ERK1/2 activation and neuritogenesis via intracellular reactive oxygen species production in human neuroblastoma SH-SY5Y cells. *Biochimica et Biophysica Acta (BBA)—Molecular and Cell Biology of Lipids*.

[50] Sun H., He X., Liu C. (2017). Effect of oleracein E, a neuroprotective tetrahydroisoquinoline, on rotenone-induced Parkinson’s disease cell and animal models. *ACS Chemical Neuroscience*.

